# Retraction: Curcumin improves age-related and surgically induced osteoarthritis by promoting autophagy in mice

**DOI:** 10.1042/BSR-2017-1691_RET

**Published:** 2022-05-30

**Keywords:** autophagy, curcumin, Osteoarthritis

This Retraction follows an Expression of Concern relating to this article previously published by Portland Press.

This article is being retracted from *Bioscience Reports* at the request of the Editor-in-Chief and the Editorial Board following receipt of a notification from a reader alerting the Editorial Board to several duplications, including between the Figure 1A 40x Curcumin and Figure 2A 100x Sham panels, the Figure 1A 40x Ctrl and Figure 2A 40x Curcumin panels, and the Figure 1A 100x Curcumin panel and the Figure 2A 40x Sham panels. The authors have been contacted with regards to the retraction and have not responded to the Journal's queries and the concerns raised. Given the extent of the issues raised, the Editorial Board stand by the decision to retract the article. The authors did not respond and therefore do not agree to the Retraction.

